# Cerebral microbleeds are not associated with postoperative delirium and postoperative cognitive dysfunction in older individuals

**DOI:** 10.1371/journal.pone.0218411

**Published:** 2019-06-14

**Authors:** Gunnar Lachmann, Ilse Kant, Florian Lammers, Victoria Windmann, Claudia Spies, Saya Speidel, Friedrich Borchers, Daniel Hadzidiakos, Jeroen Hendrikse, Georg Winterer, Jeroen de Bresser, BIOCOG Consortium

**Affiliations:** 1 Department of Anesthesiology and Operative Intensive Care Medicine (CCM, CVK), Charité–Unversitätsmedizin Berlin, Berlin, Germany; 2 Berlin Institute of Health (BIH), Berlin, Germany; 3 Department of Radiology, University Medical Center Utrecht, Utrecht, The Netherlands; 4 Department of Radiology, Leiden University Medical Center, Leiden, The Netherlands; George Washington University, UNITED STATES

## Abstract

**Background:**

Cerebral microbleeds (CMB) occur in the context of cerebral small vessel disease. Other brain MRI markers of cerebral small vessel disease are associated with the occurrence of postoperative delirium (POD) and postoperative cognitive dysfunction (POCD), but for CMB this is unknown. We aimed to study the association between CMB and the occurrence of POD and POCD in older individuals.

**Methods:**

The current study consists of 65 patients (72±5 years) from the BIOCOG study, which is a prospective, observational study of patients who underwent an elective surgery of at least 60 minutes. Patients in the current study received a preoperative cerebral MRI scan including a 3D susceptibility-weighted imaging sequence to detect CMB. The occurrence of POD was screened for twice a day until postoperative day 7 by using the DSM-5, NuDesc, CAM, and CAM-ICU. The occurrence of POCD was determined by the reliable change index model at 7 days after surgery or discharge, respectively, and 3 months after surgery. Statistical analyses consisted of logistic regression adjusted for age and gender.

**Results:**

A total of 39 CMB were detected in 17 patients (26%) prior to surgery. POD occurred in 14 out of 65 patients (22%). POCD at 7 days after surgery occurred in 11 out of 54 patients (20%) and in 3 out of 40 patients at the 3 month follow-up (8%). Preoperative CMB were not associated with the occurrence of POD (OR (95%-CI): 0.28 (0.05, 1.57); p = 0.147) or POCD at 7 days after surgery (0.76 (0.16, 3.54); p = 0.727) or at 3 months follow-up (0.61 (0.03, 11.64); p = 0.740).

**Conclusion:**

We did not find an association between preoperative CMB and the occurrence of POD or POCD.

**Trial registration:**

clinicaltrials.gov (NCT02265263) on 23 September 2014.

## Introduction

Postoperative delirium (POD) and postoperative cognitive dysfunction (POCD) occur commonly, particularly in older patients who have multimorbidity. Delirium in general is an acute condition characterized by a deterioration of attention, cognition and awareness that cannot be fully accounted for by a pre-existing neuropsychiatric disease. It might be associated with disturbances of arousal, sleep-wake cycle and affection [1]. Occurrence of POD is associated with a poor outcome and an increased risk of POCD [2, 3]. POCD consists of impairments in memory and executive functioning after surgery that tend to persist over time [4]. POD and POCD have a large incidence (POD (15–53%); POCD (10–54%)) and are currently clinically more recognized due to an increased awareness [5, 6]. Both POD and POCD are associated with a reduced quality of life, longer hospital stay, increased mortality and higher healthcare costs [7].

The exact underlying pathophysiological mechanisms of POD and POCD are still unclear, but certain structural brain changes on magnetic resonance imaging (MRI) are associated with an increased risk [8]. For instance, MRI markers of cerebral small vessel disease like white matter hyperintensities (WMH) are associated with the occurrence of POD and POCD [8]. However, for other MRI markers of cerebral small vessel disease, especially cerebral microbleeds (CMB), this is still unknown [8]. CMB occur in the context of aging and in cerebral small vessel disease and they are associated with an increased risk of cognitive decline and dementia [9]. We therefore aimed to study the association between CMB and the occurrence of POD and POCD in older patients.

## Material and methods

### Study participants

The Biomarker Development for Postoperative Cognitive Impairment in the Elderly (BIOCOG) study is a large prospective multicenter observational study, which aims to establish valid biomarkers for risk analysis and clinical outcome prediction of POD and POCD in a sample of elderly (≥65 years) patients presenting for major elective surgery (>60 min duration) at the Charité—Universitätsmedizin Berlin, Germany [10]. Further inclusion criteria were European descent, the ability to give informed consent and to undergo neuropsychological testing, and eligibility for MRI. Patients were excluded when the Mini-Mental-State-Examination was ≤23 points, when homeless or the patient would not be reachable for follow-up, when participating in another prospective interventional clinical study during hospital stay, when accommodated in an institution due to an official or judicial order, in case of neuropsychiatric morbidity, anacusis or hypoacusis, intake of centrally acting medication or any other condition which could interfere with neurocognitive testing. Overall, 1033 patients were included into the BIOCOG study. Within the BIOCOG study, our substudy was performed consisting of 66 patients who received a 3D susceptibility-weighted imaging (3D SWI) sequence in their preoperative MRI scan. Our substudy was performed in consecutive included patients between April 2016 and October 2017. One patient was excluded due to missing primary endpoints of the BIOCOG study, leaving 65 patients for our current study.

The study was approved by our medical ethics committee (Ethikkommission der Charité–Universitätsmedizin Berlin, EA2/092/14) and all patients signed an informed consent form. This clinical trial meets the requirements set out by the ICH-GCP and Declaration of Helsinki.

### Clinical data

Baseline patients demographics (age, gender, body mass index (BMI), American Society of Anesthesiologists (ASA) score, Mini-Mental State Examination (MMSE)) and cardiovascular risk factors (hypertension, diabetes, hypercholesterolemia, history of stroke, coronary and chronic heart disease) were obtained at the day of inclusion at a pre-surgery interview and by viewing the medical records. Medical history data were collected by either study physicians (anesthesiologist or anesthesiologist in training) or additional trained research staff (study nurses, psychology or MD students) under supervision of a physician. Clinical history and long-term medication data were assessed in a structured interview. Medical records were reviewed for additional data whenever a patient had presented in the clinic before. Whenever possible, records of medical findings and physician's letters were obtained from the patients and screened for additional data. Post-hoc, all clinical data have been reviewed and were validated by a study physician (anesthesiologist).

All patients received a preoperative neurocognitive assessment by trained medical staff which consisted of a CANTAB battery [11] (Paired Associates Learning, Verbal Recognition Memory, Simple Reaction Time, Spatial Span Time) as well as Trail Making Tests and Grooved Pegboard.

Peri- and postoperative parameters (surgical time, type of surgery, postoperative complications, intensive care unit (ICU) and in-patient duration, in-patient deaths) were documented by trained medical staff.

### POD and POCD

Detection of POD was conducted by screening of the patients twice a day until postoperative day 7 or discharge using the 5th edition of the Diagnostic and Statistical Manual of Mental Disorders (DSM-5) [1], NuDesc [12], CAM [13], and CAM-ICU [14]. POD is defined according to DSM-5 criteria. Patients were considered delirious in case of ≥ 2 cumulative points on the NuDesc and/or positive CAM score and/or positive CAM-ICU score and/or patient chart review that shows description of delirium (e.g., confused, agitated, drowsy, disorientated, delirious, received antipsychotic therapy).

**Fig 1:**
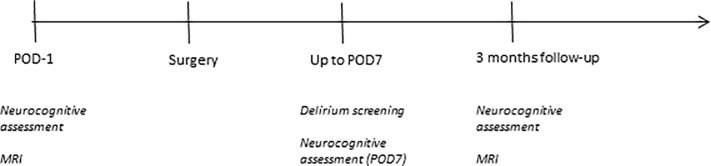
Time line of measurements.

At postoperative day 7 or discharge, respectively, as well as three months after surgery, patients received a follow-up neurocognitive assessment to detect POCD (Fig 1), which was calculated for the whole BIOCOG cohort by the reliable change index model as published by Rasmussen et al. [15]. This method corrects for learning effects and natural variability in repetitive cognitive testing by use of data from the BIOCOG non-surgical control group (n = 114), that also served to provide normative data. We imputed missing data according to random forest (technical, organizational or physiological problems) or worst case imputation paradigm (signs of cognitive impairment) whenever parts of the cognitive testing were not performed [16].

### MRI scans

MRI scans were performed at the Berlin Institute for Advance Neuroimaging (BCAN), Germany. Scans were acquired in one TIM Trio 3T MRI (Siemens, Erlangen, Germany) with a 32-channel head coil. All patients received a preoperative brain MRI scan with a standardized scan protocol including a 3D susceptibility weighted gradient echo MRI sequence (SWI, voxel size: 0.7×0.6×1.2 mm; field of view: 230×180 mm in 120 transversal slices; TE: 20 ms; TR: 28 ms, 15° flip angle) to detect CMB, a fluid-attenuated inversion recovery (FLAIR) sequence (TR/TE/inversion time = 4800/388/1800 ms; voxel size = 0.49x0.49x1.00mm^3^) and a 3D T1 magnetization-prepared rapid acquisition gradient echo sequence (TR/TE = 2500 / 4.77 ms; voxel size = 1.00 x 1.00 x 1.00 mm^3^) to detect presence of lacunar infarcts and WMH. Additionally, patients had a follow-up MRI scan 3 months after surgery with an identical scan protocol.

### MRI processing steps

CMB and presence of lacunar infarcts were determined by a (neuro-)radiologist (JB) who was blinded to patient outcomes. CMB were further categorized as lobar or deep CMB according to the Microbleed Anatomical Rating Scale [17]. Presence of lacunar infarcts was determined according to the STRIVE criteria [18]. WMH volume was determined by an automated method: the lesion prediction algorithm [19] of the lesion segmentation toolbox (LST) version 2.0.15 (www.statistical-modeling.de/lst.html), for statistical parametric mapping software (SPM12, Wellcome Institute of Neurology, University College London, UK, http://www.fil.ion.ucl.ac.uk/spm/doc/), running on Matlab version R2016b. If present, cortical infarcts were manually segmented and subtracted from the WMH probability maps before calculating the WMH volume. All WMH probability maps were checked for accurate segmentation by a trained researcher (IK).

### Statistical analysis

Results for normally distributed data are presented as arithmetic mean ± standard deviation (SD) and for non-normal distributed data as median with quartiles. Categorical data are summarized as frequencies in %. Differences in baseline patient demographics were assessed using Fisher’s exact tests for category variables, and Mann-Whitney U tests for contiguous data. Logistic regression models adjusted for age and sex were used to determine the odds ratio’s for occurrence of POD and POCD in patients with versus without preoperative CMB. Similar analyses were used for postoperative CMB patients. In exploratory analyses for POD and POCD, we performed the logistic regression models separately for lobar and deep CMB. Odds ratios (OR) are shown with 95%-confidence intervals (CI). A two-tailed p-value <0.05 was considered statistically significant. All statistical analyses were performed with IBM SPSS Statistics, Version 25.

## Results

### Study population

A total of 17 out of 65 patients (26%) had 39 CMB prior to surgery. Of these, 13 patients showed only lobar CMB, 2 patients had only deep CMB and both types of CMB were seen in 2 patients (S1 Table). Baseline patient demographics, peri- and postoperative parameters and cardiovascular risk factors did not differ between the patients with preoperative CMB versus patients without preoperative CMB (Table 1). Types of surgery are shown in S2 Table. Two patients received spinal anesthesia, all other patients were put under general anesthesia. Out of the 65 included patients, all had POD screening, 54 (83%) had a follow-up neurocognitive assessment for POCD at 7 days and 40 (62%) at 3 months after surgery. Results of the cognitive tests at baseline and after three months are presented in the S1 Text. A total of 34 patients (52%) received a follow-up brain MRI scan at 3 months. WMH volumes (p = 0.002) were significantly higher in patients with CMB.

**Table 1:** Patient characteristics and peri- and postoperative parameters.

	Total (n = 65)	Patients without CMB (n = 48)	Patients with CMB (n = 17)	P value
Demographics				
Age [years]	72.2 ± 5.2	71.7 ± 5.2	73.7 ± 5.0	0.162
Male gender	30 (46%)	23 (48%)	7 (41%)	0.779
Body Mass Index [kg/m^2^]	26.8 ± 3.9	26.9 ± 3.8	26.6 ± 4.3	0.314
**ASA score**				**0.466**
I	1 (2%)	0	1 (6%)	
II	41 (63%)	33 (69%)	8 (47%)	
III	22 (34%)	14 (29%)	8 (47%)	
IV	0	0	0	
V	1 (2%)	1 (2%)	0	
Baseline MMSE	29 (28, 30)	29 (27, 30)	29 (28, 30)	0.760
Peri- and postoperative parameters				
Surgical time [min]	105 (67, 208)	106 (71, 214)	104 (60, 173)	0.502
Intra-abdominal/-thoracic surgery	16 (25%)	13 (27%)	3 (18%)	0.528
ICU duration [days]	0 (0, 0)	0 (0, 0)	0 (0, 1)	0.724
In-patient duration [days]	7 (4, 9)	7 (4, 9)	6 (4, 10)	0.851
Deceased during hospital stay	2 (3%)	2 (4%)	0	1.000
Cardiovascular risk factors				
Hypertension	41 (63%)	28 (58%)	13 (77%)	0.247
Stroke in history	6 (9%)	3 (6%)	3 (18%)	0.179
Diabetes	14 (22%)	11 (23%)	3 (18%)	0.745
Coronary and chronic heart disease	10 (15%)	6 (13%)	4 (24%)	0.434
Hypercholesterinemia	25 (39%)	15 (31%)	10 (59%)	0.080
MRI markers of cerebral small vessel disease				
WMH volume [ml]	2.1 (0.7, 5.0)*	1.7 (0.5, 3.8)*	6.4 (1.4, 20.2)	**0.002**
Patients with lacunar infarcts	10 (15%)	5 (10%)	5 (29%)	0.111

Continuous variables in mean ± standard deviation (normal distributed data) and median (25%-75% percentiles (non-normal distributed data), frequencies with n (%); ASA, American Society of Anesthesiologists; ICU, Intensive Care Unit; MMSE, Mini-Mental State Examination; WMH, white matter hyperintensities.

*one patient had to be excluded from analysis due to previous neurosurgery.

### The association of preoperative CMB and occurrence of POD and POCD

POD occurred in 14 out of 65 patients (22%), POCD at 7 days after surgery in 11 out of 54 patients (20%) as well as in 3 out of 40 patients at the 3 month follow-up (8%). Preoperative CMB were not associated with the occurrence of POD (OR (95%-CI): 0.28 (0.05, 1.57); p = 0.147) or POCD at 7 days after surgery (0.76 (0.16, 3.54); p = 0.727) or at 3 months follow-up (0.61 (0.03, 11.64); p = 0.740) (Table 2). In exploratory analysis considering lobar and deep CMB separately, no associations were found with POD or POCD at 7 days or 3 months after surgery.

**Table 2:** The association between preoperative CMB and occurrence of POD and POCD.

	Patient without CMB (n = 48)	Patients with CMB (n = 17)	OR (95% CI)	P value
POD	12 (25%)	2 (12%)	0.278 (0.049, 1.565)	0.147
POCD 7 days after surgery*	8 (21%)	3 (19%)	0.761 (0.164, 3.535)	0.727
POCD 3 months after surgery*	2 (7%)	1 (10%)	0.606 (0.032, 11.637)	0.740

*calculated for 54 patients with POCD assessment after 7 days and 40 patients with POCD assessment after 3 months. Frequencies with n (%); CI, confident interval; OR, odds ratio; POD, postoperative delirium; POCD, postoperative cognitive dysfunction.

### Patients with preoperative CMB and new CMB after surgery

Of the 34 patients with follow-up MRI scans, 11 patients had preoperative CMB, whereas 4 of these patients (36%) developed new CMB after surgery (S1 Table). No new CMB occurred after surgery in patients without preoperative CMB. Postoperative presence of CMB was not significantly associated with POD (1.15 (0.08, 17.43; p = 0.918) or POCD at 7 days (2.66 (0.26, 27.35); p = 0.412) or at 3 months (0.34 (0.01, 8.53); p = 0.512) after surgery.

## Discussion

Our study showed that the presence of preoperative or postoperative CMB was not associated with occurrence of POD or POCD in older individuals. Our study is the first to investigate this association.

CMB are one of the MRI markers for cerebral small vessel disease [20]. Only few previous studies have examined brain MRI markers of cerebral small vessel disease in relation to POD or POCD, and to the best of our knowledge, no study has yet analyzed CMB in post-operative cognitive disorders. A recent review summarized studies investigating associations between POD/POCD and WMH/lacunar infarcts, which are other markers of cerebral small vessel disease [8]. They reported on six studies on the association between POD/POCD and WMH with a total of 504 participants that yielded contradictory results [21–26]. Especially the largest study with 146 participants did not report a significant association between WMH and POD [27]. Previous studies with a total of 71 participants on preoperative WMH and POCD have shown that WMH were related to POCD [23, 24]. No previous studies have assessed presence of lacunar infarcts as a separate measure. These results have suggested that brain MRI markers for cerebral small vessel disease might play a role in the underlying pathophysiological mechanisms of POD and POCD.

Our findings show that neither preoperative nor (new) postoperative CMB were associated with occurrence of POD or POCD. Preoperative CMB might therefore not play a role in the pathogenesis of POD or POCD. However, previous studies did show an association between other brain MRI markers of cerebral small vessel disease and POD or POCD [8]. Possible explanations for these discrepancies might be that other markers of cerebral small vessel disease are stronger preoperative predictors of POD and POCD risk. Other factors that might have played a role are our limited sample size, reducing the power to detect an association, and the relatively low prevalence of CMB in comparison to other markers for cerebral small vessel disease, such as presence of WMH. The prevalence of CMB was 26% in our cohort, which is somewhat higher compared to population based cohorts like the Rotterdam Study (19% [26]) and the Framingham Heart Study (8% [28]). However, it should be taken into account that our patients were approximately one decade older than patients in the Rotterdam study and Framingham study, while age constitutes a risk factor for CMB [29].

To the best of our knowledge, no previous studies have assessed the relation between CMB progression and occurrence of POD or POCD. However, one previous study has assessed the relation between progression of other markers of cerebral small vessel disease (WMH and infarcts) and occurrence of POCD [25]. They have shown that progression of WMH and lacunar infarcts after surgery was not related to postoperative cognitive status. Nevertheless, Patel and colleagues investigated a sample of patients presenting for cardiac surgery. New infarcts after cardiac surgery are thought to be of thrombembolic origin, although pre-operative atherosclerotic burden has been suggested to increase the risk for new post-operative brain infarcts [30]. On the other hand, intraoperative hypoperfusion is thought to cause perioperative infarcts in patients with preexisting cerebral small vessel disease, which might be similar for surgical procedures other than cardiac surgery [31]. Furthermore, we have found no new CMB after surgery in patients without preoperative CMB, but 4 out of 11 patients with preoperative CMB developed new CMB after surgery. Within these patients, it is unknown whether progression of CMB is related to progression of cerebral small vessel disease over time or is related to the operation.

Strengths of our study are the pre- and postoperative performed brain MRI scans that enabled us to systematically analyze pre- and postoperative CMB. Furthermore, the assessment of POD was done twice daily after surgery, included multiple screening tools and a chart review, which has increased our sensitivity to detect POD. We have assessed POCD according to the latest guidelines as proposed by Rasmussen et al. [15]. A limitation of our study might be the relatively small number of patients as our study was performed as a substudy. It may further be population biased as only a relatively small number of patients of the overall BIOCOG cohort were analyzed. This might reduce the power to detect an association between CMB and occurrence of POD or POCD. However, there are no previously published studies addressing the association between CMB and the occurrence of POD or POCD. Another limitation might be the relatively low number of participants at the 3 month follow-up for the assessment of POCD (62%) and for the brain MRI (52%). Also, the patients who were lost to follow-up might have been suffering from a worse postoperative cognitive and physical status compared to the patients that returned for follow-up. This could have resulted in some selection bias. Finally, one should consider that patients in our sample have rarely been admitted to the ICU for more than 24 hours. Thus, overall post-operative physical stress in our sample was probably low. This might reflect a relatively low incidence of postoperative triggers for cognitive disorders in our study. The majority of patients in our study might have not developed POD or POCD due to the lack of triggers. Taken together, these factors might have led to an underestimation of the effect of CMB on occurrence of POD or POCD.

In conclusion, we did not find an association between preoperative CMB and the occurrence of POD or POCD.

## Supporting information

S1 Table Distribution of pre- and postoperative counts of CMB. (DOCX)

S2 Table Types of surgery. (DOCX)

S1 Text Results of the cognitive tests at baseline and after three months. (DOC)

S1 Fig Results of Simple Reaction Time (SRT), Paired Associate Learning (PAL) and Simple Span Length by diagnosis. Inlays correspond to results from the repeated measures ANOVA.
(JPG)

S2 Fig Results of the Verbal Recognition Memory test (VRM) by diagnosis. Inlays correspond to results from the repeated measures ANOVA.
(JPG)

S3 Fig Results of the Trail Making Test Pt. B (TMT-B) and Grooved Pegboard Test (GPT) by diagnosis. Inlays correspond to results from the repeated measures ANOVA.
(JPG)
