# Supplementary material for: Cerebral microbleeds are not associated with postoperative delirium and postoperative cognitive dysfunction in older individuals
Source: PLoS One. 2019 Jun 14;14(6):e0218411. doi: 10.1371/journal.pone.0218411 (PMC6568413; doi:10.1371/journal.pone.0218411)
Supplement: S1 Table — (DOCX) [file pone.0218411.s001.docx]

| **Patients** | **Preoperative CMB** | **Postoperative CMB** |
| --- | --- | --- |
| 1 | 10 (lobar) | 12 (lobar) |
| 2 | 5 (lobar/deep) | 5 (lobar/deep) |
| 3 | 5 (lobar) | 5 (lobar) |
| 4 | 3 (lobar/deep) | 5 (lobar/deep) |
| 5 | 2 (lobar) | 3 (lobar) |
| 6 | 1 (lobar) | 3 (lobar/deep) |
| 7 | 1 (lobar) | 1 (lobar) |
| 8 | 1 (lobar) | 1 (lobar) |
| 9 | 1 (lobar) | 1 (lobar) |
| 10 | 1 (lobar) | 1 (lobar) |
| 11 | 1 (lobar) | 1 (lobar) |
| 12 | 1 (lobar) | -* |
| 13 | 1 (lobar) | -* |
| 14 | 2 (lobar) | -* |
| 15 | 1 (deep) | -* |
| 16 | 2 (lobar) | -* |
| 17 | 1 (deep) | -* |

*Only patients with CMB shown. Rows represent counts of CMB in each single patient. *No MRI scan due to loss to follow-up.*
