# Supplementary material for: Cerebral microbleeds are not associated with postoperative delirium and postoperative cognitive dysfunction in older individuals
Source: PLoS One. 2019 Jun 14;14(6):e0218411. doi: 10.1371/journal.pone.0218411 (PMC6568413; doi:10.1371/journal.pone.0218411)
Supplement: S2 Table — (DOCX) [file pone.0218411.s002.docx]

| **Types of surgery** |
| --- |
| Hemihepatectomy 4x  Debulking surgery for ovarian cancer 2x  Pylorus-preserving pancreaticoduodenectomy 3x  Laparoscopic hysterectomy and adnectomy 2x  Laparoscopic IPOM  Orbita biopsy  Decompression L4/5 + Sacrum biopsy  Decompression L2-4 3x  Sequestrectomy  Parathyroidectomy  Spondylodesis C3-6  Orbita excision  Tooth extraction  Total knee arthroplasty 12x  Lumpectomy 2x  Femoral-popliteal bypass  Laparoscopy and HIPEC in mesothelioma  Axillary cancer removal  Laparoscopic parotidectomy  Penile implant change  Total hip arthroplasty 8x  Prostatectomy 4x  FESS  Bladder resection  Open kidney tumor excision  Laser ablation rhinophyma  VATS + atypical resection 2x  Sacropexy 3x  Ureterorenoscopy  Laminectomy C4-7  Tracheoscopy |
