# Supplementary material for: Cerebral microbleeds are not associated with postoperative delirium and postoperative cognitive dysfunction in older individuals
Source: PLoS One. 2019 Jun 14;14(6):e0218411. doi: 10.1371/journal.pone.0218411 (PMC6568413; doi:10.1371/journal.pone.0218411)
Supplement: S1 Text — (DOC) [file pone.0218411.s003.doc]

Baseline cognitive performance for the whole sample is summarised with median, interquartile and full range. Test performance for patients without cognitive complications, POD and POCD have been summarised with mean and standard deviation.

Repeated measures ANOVA was conducted to test for effects of time, cognitive disorder (POD, POCD) and interaction. SRT latency and time for TMT-B and GPT were log-transformed prior to statistical analysis.

Table S-I summarises cognitive performance in the complete sample. Table S-II summarises baseline test performance in patients with and without postoperative cognitive disorders. Figures S1-S3 display cognitive performance in each test before and after surgery. Significant effects of time, diagnosis or an interaction were found spuriously. TMT-B performance differed between patients without and with POCD (F_1,32_=6.3, p=0.018). The number of correctly recognised items in the VRM was lower at follow-up (F_1,38_=4.4, p=0.043). GPT performance was worse in POD patients at follow-up (F_1,38_=5.3, p=0.028).

| Table S-I: Baseline cognitive function in the complete sample | | | | |
| --- | --- | --- | --- | --- |
|  | n | Median | Interquartile range | Min.-max. range |
| SRT (latency in ms for correct trials) | 64 | 299 | 258-367 | 197-904 |
| VRM (correct items in immediate free recall) | 64 | 6 | 5-8 | 2-11 |
| VRM (correct recognized items in delayed recall) | 63 | 22 | 20-23 | 14-24 |
| Span length (items) | 65 | 5 | 4-5 | 3-8 |
| PAL (first trial memory score) | 63 | 13 | 10-16.5 | 0-26 |
| GPT (completion time in s) | 61 | 89 | 76-106 | 53-250 |
| TMT-B (completion time in s) | 60 | 110 | 84-142 | 58-294 |

| Table S-II: Mean and standard deviation for cognitive test performance at baseline in patients with POD, POCD and without cognitive disorder | | | |
| --- | --- | --- | --- |
|  | No cognitive disorder  (n=49) | POD (n=14) | POCD  (n=3) |
| SRT (latency in ms for correct trials) | 314±82 | 301±79 | 323±55 |
| VRM (correct items in immediate free recall) | 6.4±2.3 | 5.7±2.4 | 5.7±1.2 |
| VRM (correct recognized items in delayed recall) | 21.8±2.3 | 21.3±2.2 | 21.3±2.9 |
| Span length (items) | 5.1±1.0 | 4.6±0.9 | 3.7±1.2 |
| PAL (first trial memory score) | 14±5 | 12±5 | 10±2 |
| GPT (completion time in s) | 89±19 | 98±21 | 105±4 |
| TMT-B (completion time in s) | 113±37 | 106±32 | 211±73 |

The BioCog study used the MMSE to exclude subjects with pre-existing cognitive deficit from enrollment. Although its wide acceptance as a dementia screening tool, the MMSE is not generally recommended as a diagnostic criterion for dementia (Folstein et al. 1975, Tombaugh and McIntyre 1992). It has further been criticized for not excluding patients with probable or mild dementia at values of 26 to 29 points (Perneczky et al. 2006). Based on analysis of cognitive test data, we compared performance in our sample with reference data to estimate the prevalence of preexisting cognitive deficits in our sample.

Compared to reference data of the age group 70-74 years presented by Tombaugh (2004), median performance in our cohort (109.86s) corresponded to the 30-40. percentiles (105-112s) in the general population, suggesting that we investigated a below-average performing population. Holtzer and colleagues presented TMT-B normative data stratified for prevalent or subsequent development of dementia and loss to follow-up. Median performance in our sample was better than mean performance in the robust cohort without subsequent dementia development (148.9s), but performance in our lowest performing cohort (142.49-293.53s) complied with mean performance in subgroups with prevalent (207.5s) or subsequent diagnosis of dementia (186.2s). In our sample, mean TMT-B performance in patients without postoperative cognitive disorder (112.7s) or POD (106.1s) was thus better than expected from Holtzer’s normative data on robust subjects. Nevertheless, patients with POCD already performed much slower at baseline (210.8s), complying with Holtzer’s reference value for demented patients.

Thus, we cannot assume that we have excluded with pre-existing mild cognitive deficits from our study. Nevertheless, since POCD in our study was derived from the reliable change index by Rasmussen, its is by definition independent of patients’ baseline performance. Furthermore, our sample represents a wide spectrum of age-related cognitive decline found in the general population. We thus assume that selection bias and ceiling effects in cognitive performance (by enrolling only high-performing subjects willing to undergo the strenuous neuroimaging and testing procedures) are not relevant factors leading to our negative result.

References:

Folstein, M.F., Folstein, S.E., McHugh, P.R., 1975. “Mini-mental state”. A practical method for grading the cognitive state of patients for the clinician. J Psychiatr Res 12, 189–198.

Holtzer, R., Goldin, Y., Zimmerman, M., Katz, M., Buschke, H., Lipton, R.B., 2008. Robust norms for selected neuropsychological tests in older adults. Archives of Clinical Neuropsychology 23, 531–541. https://doi.org/10.1016/j.acn.2008.05.004

Perneczky, R., Wagenpfeil, S., Komossa, K., Grimmer, T., Diehl, J., Kurz, A., 2006. Mapping scores onto stages: mini-mental state examination and clinical dementia rating. Am J Geriatr Psychiatry 14, 139–144. https://doi.org/10.1097/01.JGP.0000192478.82189.a8

Tombaugh, T.N., 2004. Trail Making Test A and B: Normative data stratified by age and education. Archives of Clinical Neuropsychology 19, 203–214. https://doi.org/10.1016/S0887-6177(03)00039-8

Tombaugh, T.N., McIntyre, N.J., 1992. The mini-mental state examination: a comprehensive review. J Am Geriatr Soc 40, 922–935.

Figures (separate files):

S 1: Results of Simple Reaction Time (SRT), Paired Associate Learning (PAL) and Simple Span Length by diagnosis. Inlays correspond to results from the repeated measures ANOVA.

S 2: Results of the Verbal Recognition Memory test (VRM) by diagnosis. Inlays correspond to results from the repeated measures ANOVA.

S 3: Results of the Trail Making Test Pt. B (TMT-B) and Grooved Pegboard Test (GPT) by diagnosis. Inlays correspond to results from the repeated measures ANOVA.
